# Controlling false positive rates in research and its clinical implications

**DOI:** 10.1590/2176-9451.19.3.024-025.ebo

**Published:** 2014

**Authors:** Rafael Simas, Felipe Maestri, David Normando

**Affiliations:** 1 Masters student in Dentistry, Federal University of Pará.; 2 Specialist in Orthodontics, Brazilian Dental Association - Southern Maranhão.; 3 Professor, School of Dentistry, Federal University of Pará.

Statistical analysis is, in fact, an error analysis. A statistical test does not guarantee reliable results, it only quantifies the probability of error of a given conclusion.^1^ While reading the articles of this journal, you will find a p-value. For instance, the article by Garib et al^2^ describes the p-values for a given variable at two different moments: this p-value, also known as false-positive rate,^1^ demonstrates the probability of error when asserting that there is a difference before and after expansion.

Every research is subjected to some degree of error, given that we are not investigating an entire population, but only a fraction, a sample. For this reason, when we compare two samples undergoing different treatment procedures with a view to identifying the most efficient therapy, we will always have the chance of having reached a wrong conclusion. Therefore, the lower the p-value is, the smaller the chance of error and, as a result, the more certain we are to assure that treatment "A" is more efficient than "B".

But, how can we control a false-positive error? Initially, we have to decide on the significance level (α) we expect to establish. In Dentistry, we usually set a significance level not greater than 5% (α = 5%). Nevertheless, should we increase the number of comparisons of a given study, we increase the chances of yielding outcomes that are due just to chance and, as a consequence, finding a false-positive result. The lottery is a good example. The chances of winning are little, less than 5%. However, the more we bet, the higher our chances of winning.

In statistical tests, there is a dramatic increase in false-positive rates, in which the number of comparisons is directly proportional to the number of false-positive results, as shown in Table 1.

**Table 1:** Number of comparisons (tests) and increase in false-positive rates.

# tests	α value	FW α
1	0.05	0.05
3	0.05	0.14
6	0.05	0.26
10	0.05	0.4
15	0.05	0.54

α_fw_= 1 - (1 - α_pc_)^c^

C = # of comparisons, α_pc_ stands for error type I (0.05).

Thus, when we make several comparisons using a simple statistical test, we significantly increase the chances of yielding a false-positive result. Table 1 demonstrates that the chances of yielding a false-positive result are of 40% for a study involving 10 comparisons. In these cases, some adjustments are necessary to keep the significance level set at 5%. One of the procedures employed to correct false-positive rates is the Bonferroni correction. It consists of dividing the significance level by the number of comparisons made in a given study.^3^ Suppose we carried out a comparative analysis of five cephalometric variables between two groups using an independent t-test. By dividing the significance level initially set at 0.05 or 5% by 5, the new level of error will be adjusted to 0.01 or 1%. Thus, differences will be considered significant for a p-value lower than or equal to 0.01. Nevertheless, Bonferroni correction results in a much more inflexible significance level than necessary, thus increasing the chances of yielding a false-negative rate.^4^

In 1995, Benjamini and Hochberg^5^ (BH) suggested another method to counteract false-positive rates when multiple comparisons with univariate statistical analysis are carried out. In this procedure, the researcher has to accept a minor false-positive rate and set this rate before the procedure. Suppose we compared 10 cephalometric measures between two populations A and B. After the number of comparisons is established, we determine the p-value for each analysis and organize these values in ascending order. The value of i = 1 (0.01) will be lower than the p-value, with i = 10 being the highest value. Table 2 shows the p-values in ascending order. After values are properly ranked, we apply the Benjamini-Hochberg formula: (i/m).Q (Q = false-positive acceptance rate; m = total number of comparisons). This formula allows us to correct the p-value and eliminate potential false-positive rates. With a view to obtaining the Q value, we divide the number of comparisons with P < 0.05 by the number of comparisons with P > 0.05. Table 3 shows that after finding the Q value and applying the Benjamini-Hocheberg formula, we find the corrected p-value for each comparison (i = 1, i = 2, etc.). Subsequently, we arrange the data in a table similar to Table 3, including the initial p-value and the p-value corrected by means of the formula. This method allows us to determine which comparisons are significant, in which case only those with a p-value lower than [(i/m).Q] are significant.^6^ Table 3 shows that comparisons 1 and 2 are the only ones with p-value lower than [(i/m).Q].

**Table 2:** 

Comparisons	P-value
i = 1	0.01
i = 2	0.017
i = 3	0.2
i = 4	0.22
i = 5	0.23
i = 6	0.3
i = 7	0.35
i = 8	0.4
i = 9	0.45
i = 10	0.5

**Table 3:** 

Comparisons	P-value	(i/m). Q
i = 1	**0.01**	0.025
i = 2	**0.017**	0.05
i = 3	0.2	0.075
i = 4	0.22	0.1
i = 5	0.23	0.125
i = 6	0.3	0.15
i = 7	0.35	0.175
i = 8	0.4	0.2
i = 9	0.45	0.225
i = 10	0.5	0.25

Q= 2/8 = 0.25.

In this same example, should we use Bonferroni correction to counteract error type I, comparisons 1 and 2 would probably not be significant, since α = 5% divided by the number of comparisons (ten) would result in 0.05/10 = 0.005. This value would be lower than comparisons 1 and 2 corrected by the BH technique, which demonstrates how strict Bonferroni's procedure is.

Choosing the wrong statistical test may lead clinicians to jump to conclusions. For instance, a given treatment may be considered the best one as a result of statistical analysis. Thus, statistical analysis is the key to reach more reliable clinical results. Employing more simple statistical procedures, such as the t-test, to carry out multiple comparisons, creates the need to counteract type I error (false-positive). Therefore, it is reasonable to conclude that multiple comparisons require one to carefully choose the test as well as the corrections to be employed.
